# Author’s reply to “An important step to avoid complications with leadless pacemaker implants and retrievals”

**DOI:** 10.1016/j.hrcr.2024.08.002

**Published:** 2024-08-08

**Authors:** Takehiro Nomura, Kennosuke Yamashita

**Affiliations:** Heart Rhythm Center, Department of Cardiovascular Medicine, Sendai Kosei Hospital, Miyagi, Japan

As mentioned, when retrieving a leadless pacemaker using the Aveir system, intracardiac tissue can become caught between the docking button and docking cap.^1^ Unfortunately, it is difficult to detect and predict tissue entrapment at the moment redocking occurs. However, as already indicated, the difficulty in advancing the protective sleeve can be a hint to take measures to avoid this phenomenon. We would like to emphasize again that if the protective sleeve does not smoothly advance at least half of the length of the leadless pacemaker (LP) body, the rotational handle must not be turned. If the LP is undocked before rotating it, at least severe tricuspid valve injury or cardiac perforation can most likely be avoided.

In addition, advancing the protective sleeve in the tether mode can also prevent this complication.^2^ However, in our experience, the catheter and LP might not be coaxial in the tether mode, making it difficult to advance the protective sleeve in some cases. It may be necessary to take precautions such as adequately checking the angle between the catheter and the LP under fluoroscopy.

Finally, the Aveir is currently the only dual-chamber leadless pacemaker, and the number of implantations is expected to increase in the future.^3^ Consequently, the number of retrievals is also likely to rise. It is important to climb the stairs one step at a time and not skip any steps. By sharing our experience, we sincerely hope that this complication will never happen again.
